# Modifiable Lifestyle and Environmental Determinants of Ovarian Cancer Risk: Implications for Primary Prevention

**DOI:** 10.3390/healthcare14091215

**Published:** 2026-05-01

**Authors:** Martina Arcieri, Stefano Restaino, Nicoletta Crivellaro, Giorgio Bogani, Sara Pregnolato, Doriana Armenise, Federico Paparcura, Filippo Bordin, Sara Filippin, Lino Del Pup, Lorenza Driul, Fedro Alessandro Peccatori, Carlo Ronsini, Stefano Cianci, Guglielmo Stabile, Federica Perelli, Vito Andrea Capozzi, Roberto Berretta, Giuseppe Vizzielli, The Italian Women’s Ovarian Health and Prevention Collaborative Group

**Affiliations:** 1 Clinic of Obstetrics and Gynecology, “Santa Maria della Misericordia” University Hospital, Azienda Sanitaria Universitaria Friuli Centrale, 33100 Udine, Italy; martina.arcieri@asufc.sanita.fvg.it (M.A.); giuseppe.vizzielli@uniud.it (G.V.); 2 PhD School in Biomedical Sciences, Gender Medicine, Child and Women Health, University of Sassari, 07100 Sassari, Italy; 3 Department of Medicine (DMED), University of Udine, 33100 Udine, Italy; 4 Gynaecological Oncology Unit, Fondazione IRCCS Istituto Nazionale dei Tumori di Milano, 20133 Milano, Italy; 5 Azienda Sanitaria Universitaria Friuli Centrale (ASUFC), 33100 Udine, Italy; 6 Fertility and Procreation Unit, European Institute of Oncology IRCCS, 20141 Milan, Italy; 7 Unit of Gynecologic Oncology, National Cancer Institute, IRCCS, Fondazione “G. Pascale”, 80131 Naples, Italy; 8 Obstetrics and Gynecology Unit, Department of Human Pathology of Adult and Childhood “G. Barresi”, University of Messina, 98124 Messina, Italy; 9 Department of Medical and Surgical Sciences, Institute of Obstetrics and Gynecology, University of Foggia, 71121 Foggia, Italy; 10 Pediatric Gynecology Unit, Meyer Children’s Hospital IRCCS, 50139 Florence, Italy; federica.perelli@gmail.com; 11 Azienda USL Toscana Centro, Gynecology and Obstetrics Department, Santa Maria Annunziata Hospital, 50012 Florence, Italy; 12 Department of Obstetrics and Gynecology, University of Parma, 43125 Parma, Italy

**Keywords:** ovarian cancer, primary prevention, lifestyle

## Abstract

Ovarian cancer (OC) remains the most lethal gynecologic malignancy worldwide, largely due to late-stage diagnosis and the absence of effective screening strategies. As a result, primary prevention is a critical approach to reducing disease burden. This narrative review summarizes current evidence on modifiable lifestyle, reproductive, and environmental factors associated with OC risk, based on a comprehensive PubMed, MEDLINE, Scopus, and Web of Science search conducted through April 2026. Consistent protective associations have been reported for reproductive factors, including parity, breastfeeding, oral contraceptive use, salpingectomy, and tubal ligation. Among lifestyle factors, excess body weight is modestly associated with increased OC risk, while evidence regarding physical activity remains inconclusive. Diets rich in fiber and aligned with a Mediterranean pattern appear protective, potentially through hormonal modulation and anti-inflammatory effects. In contrast, pro-inflammatory diets high in trans fats and refined carbohydrates may increase risk, whereas omega-3 fatty acids show potential protective benefits. Chronic pelvic inflammation, particularly related to Chlamydia trachomatis infection, has been linked to elevated epithelial OC risk. Smoking demonstrates a dose–response association with mucinous tumors. Environmental exposures, including genital talc use and endocrine-disrupting chemicals such as phthalates and bisphenols, have linked to a possible, albeit modest, increase in risk, although the causal mechanisms remain uncertain. Although individual associations are generally modest, their cumulative population impact may be substantial. Integrating lifestyle-based prevention strategies into gynecologic practice and public health initiatives could represent a cost-effective approach to reducing OC incidence and improving women’s health outcomes.

## 1. Introduction

Ovarian cancer (OC) is the eighth most frequently diagnosed malignancy among women worldwide and remains the leading cause of death from gynecologic cancers [1]. Despite advances in cytoreductive surgery, platinum-based chemotherapy, and the introduction of targeted maintenance therapies, long-term survival has improved only modestly, with five-year survival rates remaining below 40%. A major determinant of poor prognosis is the fact that more than 70% of cases are diagnosed at advanced stages, largely due to the asymptomatic nature of early disease and the absence of effective screening tools. Large randomized trials, including the UKCTOCS study, have demonstrated that screening strategies based on CA-125 measurement and transvaginal ultrasound do not reduce OC mortality [2]. In the absence of reliable early detection methods, primary prevention represents the most promising strategy to reduce disease incidence.

The etiology of OC is complex and multifactorial. Established non-modifiable risk factors include increasing age, inherited genetic mutations such as BRCA1 and BRCA2, and family history of ovarian or breast cancer [3,4,5]. However, these factors account for only a proportion of cases. Consequently, identifying modifiable exposures that may influence risk is critical for effective prevention strategies.

The concept of primary prevention in oncology focuses on reducing disease incidence by modifying risk factors before cancer develops. In OC, reproductive factors such as parity and oral contraceptive use have long been recognized as protective. Nevertheless, growing attention has been directed toward lifestyle and environmental determinants, including smoking, obesity, diet, hormonal exposures, and environmental chemicals, that may contribute to ovarian carcinogenesis.

Importantly, many of modifiable factors similarly influence the risk of other hormone-related malignancies, including breast and endometrial cancers, suggesting shared biological mechanisms across the female reproductive system [6].

From a public health standpoint, lifestyle-based interventions offer cost-effective, scalable strategies for cancer prevention.

This narrative review synthesizes current evidence regarding lifestyle, dietary, and environmental determinants of OC risk. By identifying modifiable exposures and estimating their preventive potential, we aim to provide an evidence-based framework to inform public health initiatives and preventive counselling in gynecologic practice.

## 2. Materials and Methods

This study was conducted as a narrative review summarizing current evidence on modifiable lifestyle and environmental factors associated with OC risk. A comprehensive search of PubMed, MEDLINE, Scopus, and Web of Science was performed from database inception through April, 2026 to identify relevant studies. Search terms included combinations of keywords such as “ovarian cancer,” “lifestyle,” “environmental exposure,” “diet,” “physical activity,” “obesity,” “diabetes,” “inflammation,” “pelvic inflammation,” “smoking,” “vitamin D,” “hormone therapy,” “oral contraceptives,” “salpingectomy,” “reproductive factors,” “environmental factors,” and “prevention.” Reference lists of selected articles were also manually screened to identify additional relevant studies.

Eligible publications included systematic reviews, meta-analyses, randomized controlled trials, and large prospective cohort studies addressing primary prevention or risk modification. Observational and case–control studies were also included when they provided significant mechanistic or epidemiologic insights. Studies focused exclusively on treatment or prognosis were excluded. We give most weight to evidence from meta-analyses and pooled analyses.

Reference lists of relevant publications and major reports from the World Cancer Research Fund and the American Institute for Cancer Research were screened to identify additional pertinent studies. Evidence was synthesized thematically according to major modifiable domains: reproductive determinants, body weight, adiposity, diabetes, physical activity, dietary patterns, vitamin D status, infections and inflammation, environmental toxins, and substance use.

Given the narrative nature of this review, no formal quality scoring or quantitative meta-analysis was performed. The methodology adhered to accepted standards for narrative epidemiologic synthesis, emphasizing transparency, reproducibility, and balanced interpretation of evidence strength.

The initial search yielded approximately 500 records. After removal of duplicates, titles and abstracts were screened for relevance. Studies were included if they evaluated associations between modifiable lifestyle or environmental exposures and OC risk. Exclusion criteria included non-English articles, case reports, editorials, and studies lacking sufficient methodological detail.

**Figure 1:**
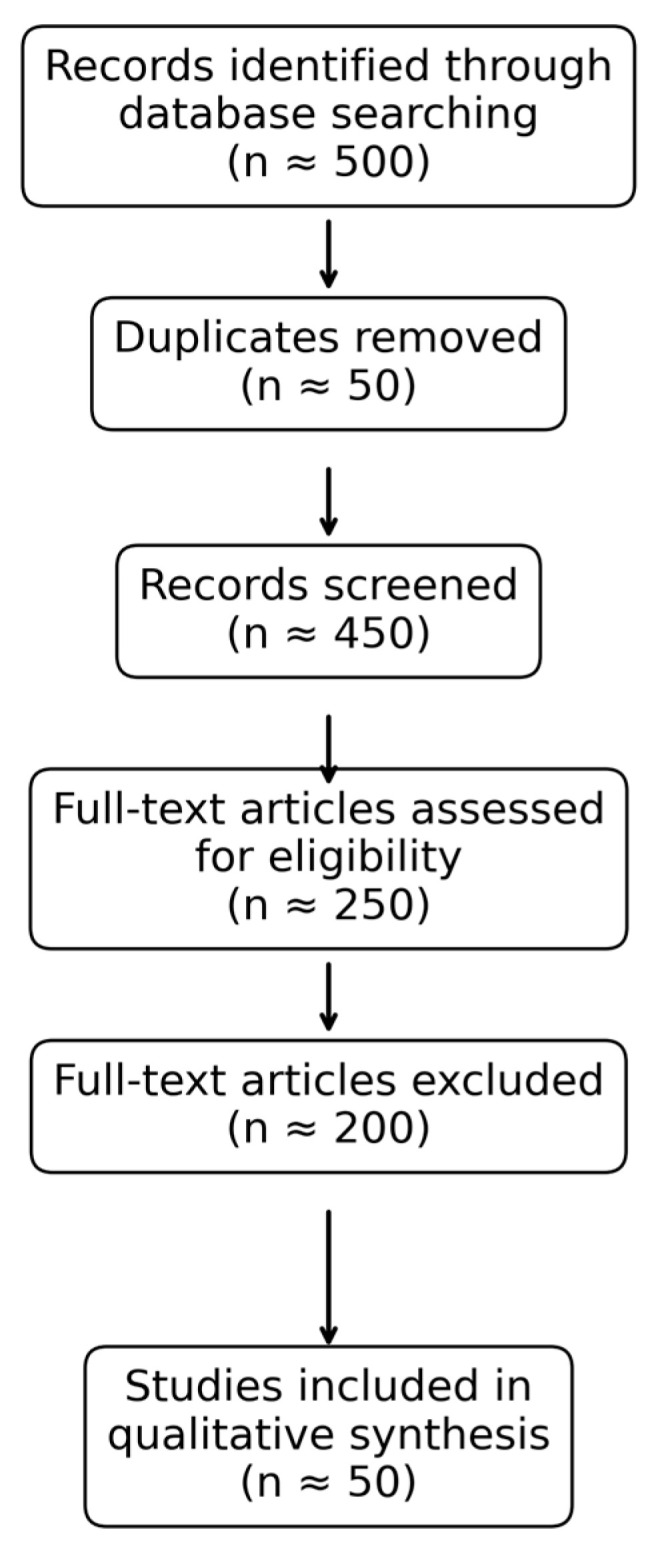
Study Selection Flow Diagram.

Full texts of potentially eligible articles 200 were reviewed, and a final set of 50 studies was included in the qualitative synthesis (Figure 1).

## 3. Results

### 3.1. Etiological Framework of Ovarian Cancer

OC comprises a heterogeneous group of malignancies with distinct histological and molecular characteristics. The most common subtype is epithelial OC, which includes serous, endometrioid, clear cell, and mucinous tumors. Differences in risk factors and pathogenesis among these subtypes complicate epidemiological investigations.

The hormonal etiology of epithelial OC is explained by two main, non-mutually exclusive hypotheses that represent the current understanding of the disease.

One of the most widely accepted is the “incessant ovulation” hypothesis, which suggests that repeated ovulation leads to cumulative damage to the ovarian epithelium, increasing the likelihood of malignant transformation. Factors that suppress ovulation, such as pregnancy and oral contraceptive use, therefore appear to reduce risk [7]. Another hypothesis is the “gonadotropin stimulation” theory, which proposes that elevated gonadotropin levels stimulate ovarian epithelial proliferation, increasing susceptibility to malignant transformation [8]. Hormonal exposures may influence this pathway.

Inflammation has also been proposed as a central mechanism in ovarian carcinogenesis. Ovulation is an inflammatory process involving cytokine release and tissue remodelling. Chronic inflammatory exposures, including smoking or environmental toxins, may exacerbate this process and contribute to malignant transformation [9].

**Figure 2:**
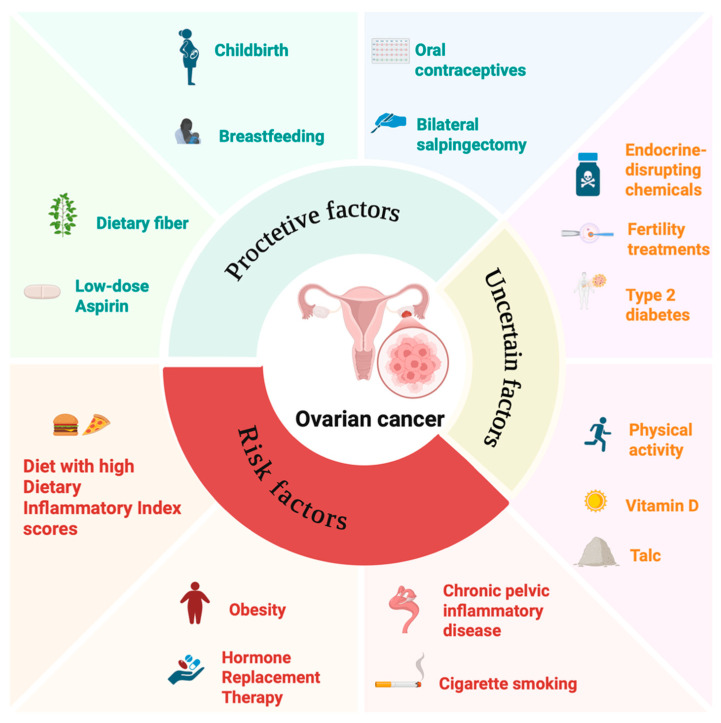
Lifestyle and Environmental Determinants of Ovarian Cancer Risk. Created in BioRender. Vizzielli, G. (2026) https://BioRender.com/wjrs2ce (accessed on 16 April 2026).

While non-modifiable factors such as genetics play an important role, a growing body of evidence suggests that lifestyle and environmental exposures may modify these biological pathways (Figure 2).

### 3.2. Reproductive Determinants

Although some reproductive factors are not strictly modifiable later in life, behavioral choices related to reproduction may influence OC risk.

Reproductive factors—such as parity, breastfeeding, and oral contraceptive use—have been consistently associated with a lower risk of OC. In particular, parity and breastfeeding are linked to a lower risk of epithelial OC, likely due to ovulation suppression and the hormonal changes occurring during pregnancy and lactation.

A meta-analysis evaluating parity found that, compared with nulliparous women, the pooled relative risks (RRs) for OC were 0.72 (95% CI, 0.65–0.79) for one birth, 0.57 (95% CI, 0.49–0.65) for two births, and 0.46 (95% CI, 0.41–0.52) for three or more births. Similarly, breastfeeding was associated with a progressive reduction in risk according to duration, including among carriers of BRCA1/2 mutations [10]. The pooled RRs were 0.79 (95% CI, 0.72–0.87) for breastfeeding for less than 6 months, 0.72 (95% CI, 0.64–0.81) for 6–12 months, and 0.67 (95% CI, 0.56–0.79) for more than 13 months. Notably, the greatest protective effect was observed with the first birth and the first six months of breastfeeding, while additional births and longer breastfeeding durations provided further, though more modest, reductions in risk. Overall, the first birth alone was associated with nearly a 30% reduction in OC risk, and the combined effect of the first birth and less than six months of breastfeeding reached approximately 40%, suggesting that breastfeeding contributes an additional risk reduction of about 10% [11].

Combined oral contraceptives are estimated to reduce OC risk by 30–50% [12,13] and appear to provide substantial protection even among younger women at elevated risk, including BRCA mutation carriers [14]. Nevertheless, their use has been associated with a slight increase in breast cancer risk [15]. A meta-analysis of 24 case–control and cohort studies reported a significantly lower incidence of OC among women who had ever used oral contraceptives compared with never users (OR 0.73; 95% CI, 0.66–0.81). The protective effect increased with longer duration of use; women who used oral contraceptives for more than 10 years had a substantially lower risk (OR 0.43; 95% CI, 0.37–0.51) [16].

An increasing body of evidence supports the hypothesis that the biological effects of progestins may represent a key mechanism underlying the protective effect against OC observed with both oral contraceptive use and pregnancy [17].

Current evidence indicates that salpingectomy is significantly associated with a reduced risk of tubo-ovarian carcinoma [18,19]. This strategy is based on the recognition that many high-grade serous OCs may originate from the distal fallopian tube rather than the ovarian epithelium itself [20]. Similarly, tubal ligation has been identified as a protective factor against epithelial OC [21]. Recent evidence suggests that bilateral salpingo-oophorectomy may be considered in postmenopausal women undergoing pelvic surgery for benign conditions, such as hysterectomy for uterine fibroids or colorectal surgery [22].

Evidence regarding the association between hormone replacement therapy (HRT) and OC risk has evolved over time. A pooled analysis reported that HRT use was associated with a modestly increased risk of OC with pooled relative risks of 1.20 (95% CI 1.01–1.44) in cohort studies and 1.13 (95% CI 1.04–1.22) in case–control studies. However, when analyses were restricted to studies conducted in more recent decades, this association was no longer statistically significant in cohort studies published after 2010 and in case–control studies after 2006, suggesting that changes in prescribing patterns and formulations may have influenced risk estimates. Both estrogen replacement therapy (ERT) and combined estrogen–progesterone replacement therapy (EPRT) were associated with only small increases in risk, although prolonged use—particularly for durations exceeding 10 years—appeared to be associated with a higher risk.

Among nonusers, hormone use for less than five years was not associated with an increased risk of OC. However, longer durations of use were linked to elevated risk, with summary estimates of 1.13 (95% CI 0.99–1.29) for more than five years and 1.37 (95% CI 1.02–1.85) for more than ten years, respectively. Subgroup analyses also suggested that serous ovarian cancer may be more sensitive to hormonal exposure than other histological subtypes. Overall, current evidence indicates that the OC risk associated with HRT may be relatively modest and has likely declined over time; however, long-term use of estrogen-only therapy may still increase risk. For women requiring prolonged therapy, continuous combined EPRT may represent a safer alternative compared with estrogen-only regimens [23]. Progesterone appears to exert anti-tumor effects in OC development [23], consistent with the protective effects observed with oral contraceptive use or pregnancy. The potential relationship between fertility drugs and OC has been widely investigated, but findings remain inconsistent. Overall, current evidence does not show a clear association between fertility drug use and invasive OC [24]. A recent meta-analysis suggested that fertility treatments may be associated with an increased risk of borderline ovarian tumors. In particular, the use of in vitro fertilization or clomiphene citrate was linked to modest increases in risk of approximately 30–40% [25]. A recent large population-based cohort study of infertile women aged 20–45 years in Denmark (1995–2017), drawn from the Danish Infertility Cohort (n = 146,110) and linked to nationwide registries, reported different findings. Over a median follow-up of 10.3 years, 114 women were diagnosed with OC, including 65 cases of serous OC. No associations were observed between most fertility drugs and the risk of OC. However, progesterone use was associated with an increased rate of serous ovarian cancer (HR 1.92; 95% CI 1.16–3.17), with no clear variation by time since first use or cumulative dose. Overall, these results suggest a potential link between progesterone use and an increased risk of serous OC [26], in contrast to the protective effects reported for pregnancy, EPRT, and combined oral contraceptives.

However, it remains unclear whether this association is attributable to the fertility treatments themselves or to the underlying infertility conditions [1,24].

### 3.3. Body Weight, Adiposity and Diabetes

Sustained chronic inflammation can drive carcinogenesis by damaging critical cellular components, activating tumor-promoting signaling pathways, enhancing abnormal cell proliferation, and suppressing apoptosis. Factors such as excess body weight, adiposity, and diabetes may contribute to this chronic inflammatory state.

Although many studies have explored the link between adiposity and OC, results remain heterogeneous and the overall association modest [27,28]. Elevated body mass index (BMI) is an established risk factor for several malignancies, including endometrial and postmenopausal breast cancer, yet its relationship with OC appears weaker [6,29].

A 2012 meta-analysis reported that among women who had never used HRT, each 5 kg/m^2^ increase in BMI was associated with an approximately 10% higher risk (relative risk ≈ 1.1). In contrast, no significant association was observed among HRT users, indicating that endogenous estrogen exposure may influence the relationship between adiposity and OC risk [28]. Emerging evidence indicates histotype-specific associations, with stronger links observed for borderline serous tumors, invasive low-grade serous, mucinous and endometrioid OCs. Among postmenopausal women, these associations do not appear to differ between users and non-users of HRT. Notably, obesity is not associated with an increased risk of high-grade invasive serous cancers, suggesting that reductions in BMI are unlikely to prevent the majority of OC –related deaths [30].

Evidence suggests that obesity may increase cancer risk through multiple pathways, including enhanced peripheral aromatization of androgens to estrogens, chronic low-grade inflammation, oxidative stress, hyperinsulinemia and insulin resistance, disruption of the Insulin-like growth factor I levels (IGF-1) axis, and alterations in sex hormone biosynthesis and signaling [31].

Although the strength of association is limited compared to other obesity-related cancers, maintaining healthy body weight remains a central public health priority given the global prevalence of overweight and obesity.

Evidence regarding the association between type 2 diabetes and OC risk remains inconclusive. A 2020 meta-analysis reported a statistically significant 17% increased risk of OC among women with type 2 diabetes compared with those without the condition (RR 1.20; 95% CI 1.10–1.31 for all studies), although results varied by study design, with an RR of 1.08 (95% CI 0.77–1.53) for case–control studies and 1.22 (95% CI 1.11–1.33) for cohort studies; however, substantial heterogeneity between studies was observed [32]. Chronic hyperinsulinemia may promote tumor development through the oncogenic effects of insulin, including activation of cellular signalling pathways and stimulation of growth factor-mediated proliferation. Elevated insulin levels are associated with increased bioactivity of IGF-1, which exerts mitogenic and anti-apoptotic effects on both normal and cancer cells, thereby contributing to carcinogenesis in type 2 diabetes. In addition, hyperglycemia is linked to oxidative stress, characterized by an imbalance between reactive oxygen species [33].

In contrast, more recent studies and an umbrella review concluded that the overall evidence supporting this link is weak. Part of the observed heterogeneity may be related to differences in diabetes treatments, as metformin—the most commonly prescribed first-line therapy for type 2 diabetes—has been suggested to potentially reduce OC risk. Although in vitro studies indicate possible anticancer effects of metformin, these experiments often involve doses much higher than those used clinically. Epidemiological studies investigating this relationship have produced mixed results, and many have been limited by insufficient adjustment for potential confounding factors [1].

### 3.4. Physical Activity and Sedentary Behavior

Physical activity (PA) has been associated with reduced risk of several cancers, including breast and colorectal cancer. Evidence for OC is less consistent but suggests potential protective effects. Notably, findings from prospective cohort studies often differ from those of case–control studies. While several case–control studies suggest that PA may have a protective effect against OC, this association is frequently non-significant—or even reversed—in large cohort studies. These discrepancies may be explained by recall bias in case–control designs, residual confounding, limited sample sizes, reliance on a single assessment of PA, and insufficient information on the timing, type, and intensity of activity. Furthermore, a recent meta-analysis also failed to identify a significant association between PA and OC risk. Neither moderate PA (RR = 1.04, 95% CI 0.97–1.10; *p* = 0.263) nor high levels of PA (RR = 1.04, 95% CI 0.92–1.18; *p* = 0.488) were significantly associated with OC risk [34].

Despite these inconsistencies, PA remains a modifiable lifestyle factor that could potentially reduce OC risk. Proposed mechanisms include lowering circulating estrogen levels, reducing obesity and inflammation, and improving immune function, particularly in postmenopausal women [35].

### 3.5. Dietary Habits and Nutritional Patterns

Dietary patterns have long been investigated as potential determinants of cancer risk.

Higher dietary fiber intake has been consistently associated with a reduced risk of OC. A metanalysis of observational studies found that women in the highest categories of fiber consumption show a significantly lower risk compared with those in the lowest intake group, both in case–control studies (RR = 0.75, 95%CI = 0.68–0.83) and in cohort studies (RR = 0.76, 95%CI = 0.63–0.92) [36]. Dose–response analyses further indicate that OC risk decreases by approximately 3% (RR = 0.97; 95% CI: 0.95–0.99) for every 5 g/day increase in dietary fiber intake [37].

Previous research has demonstrated that greater fiber intake is linked to lower circulating estrogen levels and reduced bioavailability of steroid hormones. Since serum estrogen can stimulate ovarian epithelial cell proliferation and promote tumor progression, it is biologically plausible that higher dietary fiber intake may be inversely related to OC risk [36]. Dietary fiber plays an important role in maintaining hormonal and metabolic balance and is a key component of the Mediterranean diet, which is characterized by high intakes of fiber, antioxidants, and other bioactive compounds. The Mediterranean diet exerts well-documented anti-inflammatory and antioxidant effects, lowersIGF-1 and has been associated with a reduced risk of several hormone-related malignancies. A recent trial highlighted that adherence to a Mediterranean diet with moderate protein restriction was associated with a significantly lower incidence of BRCA-related cancers (HR = 0.58; 95% CI: 0.35–0.96; *p* = 0.03) [38]. Taken together, adherence to a fiber-rich Mediterranean-style dietary pattern may represent a cornerstone of lifestyle-based strategies for ovarian cancer prevention.

In addition, recent evidence suggests that plant-based dietary patterns may influence ovarian cancer risk. Diets emphasizing healthy plant foods have been associated with a lower risk, whereas plant-based diets high in less healthy plant-derived foods appear to be linked to an increased risk (OR = 1.78, 95% CI: 1.40–2.28) [39].

Indeed, pro-inflammatory diet are associated with an increased risk of OC. Pro-inflammatory dietary patterns characterized by high intakes of trans fatty acids and refined carbohydrates have been associated with an increased risk of OC [40,41,42,43]. A recent meta-analysis showed that individuals with higher Dietary Inflammatory Index (DII) scores had a 42% increased risk of OC (OR = 1.42; 95% CI 1.19–1.65). Subgroup analyses indicated that this association was significant among postmenopausal women (OR = 1.72; 95% CI 1.26–2.21), but not among pre- or perimenopausal women (OR = 1.21; 95% CI 0.63–1.79) [44].

In contrast, omega-3 polyunsaturated fatty acids, particularly docosahexaenoic acid (DHA), have demonstrated anti-proliferative and pro-apoptotic effects and have been inversely associated with OC, especially endometrioid subtypes (OR = 0.58, 95% CI 0.41–0.82) [42]. Additionally, dietary patterns emphasizing complex carbohydrates and a low glycemic load may help reduce hyperinsulinemia-driven carcinogenic mechanisms. High glycemic load intake was associated with an increased risk, with an OR of 1.7 (95% CI 1.3–2.1) [45].

### 3.6. Pelvic Infections and Chronic Inflammation

Chronic pelvic inflammatory disease (PID), particularly in cases associated with recurrent Chlamydia trachomatis infection, has been associated with a heightened risk of epithelial OC [46,47,48]. A recent metanalysis showed that a history of PID was associated with significantly increased risk of developing OC (OR = 1.48; 95% CI 1.03–2.21) [47]. Histotype-specific analyses suggest a particularly strong link with serous epithelial OC (aOR = 1.46; 95% confidence interval, 1.18–1.80)—especially high-grade serous carcinoma (aOR = 1.43; 95% confidence interval, 1.01–2.04) and borderline serous OC (aOR = 1.76; 95% CI, 1.36–2.29)—and a possible association with clear cell carcinoma [49,50]. From a biological standpoint, these findings are consistent with the role of chronic inflammation in carcinogenesis. Ongoing inflammation of the fallopian tubes may facilitate carcinogenesis by disrupting cytokine regulation and inducing microenvironmental alterations that promote malignant transformation [48]. These observations underscore the importance of timely and effective management of PID. Preventive approaches include routine screening for sexually transmitted infections, early and appropriate antimicrobial therapy, and comprehensive sexual health education.

As previously mentioned, chronic inflammation plays a central role in ovarian carcinogenesis. Anti-inflammatory agents, including low-dose aspirin, may reduce this risk by inhibiting cyclooxygenase enzymes and decreasing the production of inflammatory mediators, as well as through cyclooxygenase-independent mechanisms such as modulation of Wnt/β-catenin and NF-κB signaling pathways.

A metanalysis has shown that low-dose aspirin use is associated with a reduced risk of ovarian cancer, particularly among nulliparous women. Overall, frequent aspirin use was linked to a 10% reduction in risk in cohort studies (HR 0.90; 95% CI 0.81–1.01) and a 16% reduction in case–control studies (OR 0.84; 95% CI 0.72–0.98) [51].

### 3.7. Vitamin D

Vitamin D plays an important role in regulating cellular proliferation, differentiation, and apoptosis. Epidemiological studies suggest that lower circulating levels of vitamin D are associated with an increased risk of OC [52]. Observational evidence further indicates a higher incidence of OC—particularly borderline tumors—in regions with low ultraviolet-B exposure and reduced serum 25-hydroxyvitamin D concentrations [53]. Instead, data from a meta-analysis showed no significant association between total vitamin D intake and OC risk, with a pooled RR of 1.02 (95% CI 0.89–1.16) [54]. Therefore, data regarding vitamin D supplementation and its potential preventive role in OC remain limited and inconsistent [54].

### 3.8. Tobacco

Cigarette smoking is a well-established risk factor for several cancers while its role in OC risk is more complex and appears to vary by histological subtype.

Epidemiological studies have demonstrated that smoking is associated with an increased risk of mucinous ovarian tumors (OR = 1.9; 95% CI 1.3–2.9), but it was not associated with nonmucinous tumors (OR = 1.1; 95% CI 0.9–1.3). These findings were supported by meta-analytic evidence. The overall summary RR for OC among current versus never smokers was 1.05 (95% CI 0.95–1.16), with estimates of 0.97 (95% CI 0.84–1.12) in case–control studies and 1.15 (95% CI 0.99–1.33) in cohort studies. A significantly increased risk was observed for mucinous OC (RR 1.78; 95% CI 1.52–2.07), whereas no significant association was found for serous OC (RR 1.05; 95% CI 0.94–1.17). Specifically, the RR was 1.44 (95% CI 1.23–1.67) for invasive mucinous cancer and 2.09 (95% CI 1.78–2.46) for borderline mucinous tumors (*p* = 0.039) [55]. Among women with mucinous tumors, the risk rose with greater cumulative smoking exposure: ORs were 1.0 for <5 pack-years, 1.9 for 5–24 pack-years, and 2.7 for ≥25 pack-years (*p* for trend = 0.01), indicating a clear dose–response relationship [55,56]. Nicotine and its metabolites have been detected in ovarian tissue, suggesting that these compounds may directly damage DNA in the ovarian surface epithelium. In addition, cigarette smoking has been associated with higher circulating levels of gonadotropins and androgens, both of which may adversely influence OC risk [17]. Tobacco smoking may exert a stronger influence during the early stages of ovarian carcinogenesis. The more pronounced association with mucinous tumors may reflect their developmental continuum from benign to borderline and invasive disease, whereas serous ovarian cancers are often high-grade and do not typically arise from borderline lesions. Furthermore, smoking-related somatic mutations in the KRAS gene are more frequently observed in mucinous than in serous ovarian tumors and are also more common in borderline tumors than in invasive cancers [33].

Conversely, smokers tend to experience earlier menopause, a factor that could potentially reduce risk by shortening the lifetime number of ovulatory cycles [17].

### 3.9. Environmental Exposures

Talc is a desiccant that has long been used as baby powder. Because talc deposits can occur near asbestos, concerns have been raised about potential contamination. Retrospective and case–control studies have suggested an association between genital talc use and OC; however, prospective cohort studies have not consistently confirmed these findings. Furthermore, the positive associations reported in case–control studies are generally modest (RR ≈ 1.22), particularly for serous subtypes, and the proposed causal mechanisms remain uncertain [57,58,59]. In addition, endocrine-disrupting chemicals (EDCs), including phthalates and bisphenols, may interfere with hormonal signaling pathways and potentially contribute to carcinogenesis [60]. Although the absolute increase in risk appears limited, reducing exposure to talc and known EDCs may represent a reasonable precautionary strategy.

## 4. Discussion

The evidence synthesized in this review underscores the important role of modifiable lifestyle and environmental exposures in the prevention of OC. Table 1 summarizes the positive and negative associations between these factors, as identified in the meta-analyses.

Importantly, most research evaluating reproductive, lifestyle, and demographic factors related to OC is derived from epidemiologic studies, as randomized controlled trials (RCTs) are often neither ethical nor feasible for assigning individuals to potentially harmful exposures. Moreover, RCTs are particularly challenging in this context because many relevant exposures—such as reproductive behaviors or long-term lifestyle factors—cannot be ethically or practically randomized (e.g., parity or breastfeeding), and the long latency between exposure and cancer development would require prolonged follow-up and large sample sizes. These studies generally fall into two main designs: case–control studies and cohort studies, each characterized by specific strengths and limitations. In case–control studies, information on lifestyle and related variables is typically collected within two months to one year after diagnosis. These data may include early-life exposures as well as factors occurring shortly before diagnosis. In contrast, prospective cohort studies recruit participants many years before any diagnosis of OC, with OC cases usually representing only a small proportion of the total cohort. Many cohort studies rely on a single baseline questionnaire or a limited number of follow-up assessments. Case–control studies present two primary limitations. First, recall of pre-diagnosis exposures may be subject to misclassification, as participants’ recollections could be influenced by their disease status. Second, women with the most aggressive tumors may be underrepresented due to challenges in recruitment. Despite these limitations, case–control studies often include larger numbers of OC cases and provide more detailed information on clinical characteristics and lifestyle variables that are particularly relevant to ovarian cancer. It is also important to note that participants already have the disease at the time of data collection. By comparison, cohort studies generally include relatively few OC cases, which can reduce statistical power for survival analyses. They may also lack detailed clinical data and often rely on exposure information collected many years prior to diagnosis. Nevertheless, cohort studies offer notable advantages: exposure data are obtained before disease onset, reducing the potential for recall bias, and case ascertainment is more likely to capture women with highly aggressive tumors because participants were enrolled in the cohort prior to diagnosis. However, the completeness of case identification in cohort studies may vary depending on follow-up procedures. These strengths and limitations of both study designs should be considered when interpreting findings on lifestyle factors associated with OC risk [61]. Notably, evidence suggests that, on average, pooled effect estimates from observational studies and RCTs are broadly comparable, although variability may arise due to differences in study populations, exposure definitions, and methodological approaches. These considerations should be taken into account when interpreting findings on lifestyle factors associated with OC risk [62]. In conclusion, although the magnitude of the individual associations highlighted in this review is generally modest, their cumulative population impact may be substantial.

Maintaining healthy body weight and engaging in regular physical activity provide broad oncologic and cardiometabolic benefits. Dietary patterns characterized by high fiber intake and low inflammatory potential appear protective. Chronic inflammation and infection represent emerging mechanistic drivers of ovarian carcinogenesis, reinforcing the importance of pelvic health and sexually transmitted infection prevention. Environmental exposures and substance use, particularly smoking, exhibit subtype-specific associations and warrant targeted counseling. Collectively, these findings demonstrate that OC is not entirely predetermined by genetic or reproductive factors. Multifactorial lifestyle modification may meaningfully reduce incidence. Ensuring that women are informed about how reproductive and lifestyle choices may influence their future cancer risk is therefore essential. Despite these challenges, emerging preventive strategies, particularly salpingectomy, offer promising opportunities to reduce the incidence of OC.

However, some limitations of this narrative review should be acknowledged. First, the absence of a formal systematic review protocol and quantitative synthesis may introduce selection bias and limit the reproducibility of the findings. Second, the included evidence is primarily derived from observational studies, which are inherently subject to residual confounding and variability in exposure assessment. Moreover, these studies often do not allow a precise determination of causal relationships, making it difficult to clearly distinguish correlation from causation. Third, heterogeneity across studies in terms of populations, study designs, and definitions of exposures and outcomes may affect the comparability and interpretation of results. Finally, publication bias and the selective reporting of significant findings cannot be excluded.

Despite these limitations, this review provides a comprehensive overview of current evidence and highlights key areas for future research and prevention strategies in OC.

Given that many lifestyle factors show inconsistent associations with OC risk, partly due to heterogeneity among studies, variation in histological subtypes, and the presence of confounding factors, future research should prioritize several key areas. These include large prospective cohort studies with detailed exposure assessments, investigations into the interaction between genetic susceptibility and lifestyle factors, and studies aimed at elucidating the molecular mechanisms linking lifestyle exposures to ovarian carcinogenesis. In addition, evaluating population-level prevention strategies will be essential. A better understanding of these determinants may help identify high-risk populations and support the development of more effective, targeted prevention approaches.

## 5. Conclusions

Ovarian cancer remains a major public health challenge due to late-stage diagnosis and ineffective screening. Growing evidence supports the role of modifiable lifestyle factors—including weight management, high-fiber anti-inflammatory diets, infection prevention, and avoidance of tobacco—in reducing risk.

However, most of the available evidence is derived from observational studies, which are inherently subject to residual confounding, exposure misclassification, and heterogeneity across study designs and populations. In addition, for several factors, the magnitude of associations is modest and findings remain inconsistent, limiting the ability to draw definitive causal inferences.

Integrating lifestyle-based prevention into gynecologic practice and public health strategies should therefore be considered with caution but recognized as a potentially valuable component of comprehensive cancer control. Such an approach may offer synergistic benefits across multiple hormone-related malignancies and contribute to improved women’s health outcomes at the population level, while further high-quality research is needed to clarify the strength and causality of these associations.

## Figures and Tables

**Table 1:** Associations of Risk and Protective Factors with Ovarian Cancer: Evidence from Meta-Analyses.

Category	RR/OR (95% CI)	Note	Authors
**Childbirth**	**↓ RISK** RR 0.72 (0.65–0.79) for 1 birth, 0.57 (0.49–0.65) for 2 births, and 0.46 (0.41–0.52) for ≥3 births		Sung et al., 2016 [11]
**Breastfeeding**	**↓ RISK** RR 0.79 (0.72–0.87) for breastfeeding for <6 months, 0.72 (0.64–0.81) for 6–12 months, and 0.67 (0.56–0.79) for >13 months.		Sung et al., 2016 [11]
**Oral contraceptives**	**↓ RISK** OR 0.73 (0.66–0.81) OR 0.43 (0.37–0.51) (user for >10)		Havrilesky et al., 2013 [16]
**Bilateral salpingectomy**	**↓ RISK** OR 0.48 (0.33–0.69)		Tang et al., 2025 [19]
**HRT**	**↑ RISK** RR 1.20 (1.01–1.44) from cohort studies OR 1.13 (1.04–1.22) from case–control studies RR of 1.15 (0.82–1.61) from cohort studies conducted after 2010 OR 1.09 (CI 0.93–1.27) from case–control studies conducted after 2006	The overall risk of OC has not increased among HRT users for less than 5 years	Xiang et al., 2024 [23]
**Obesity**	**↑ RISK** OR = 1.24 (1.18–1.30) for serous BOT OR 1.17 (1.11–1.23) for invasive endometrioid OC OR 1.19 (1.06–1.32) for invasive mucinous OC OR 1.13 (1.03–1.25) for low-grade serous invasive OC	Pooled analysis of 15 case–control studies	Olsen et al., 2013 [30]
**Dietary fiber**	**↓ RISK** RR 0.760 (0.702–0.823)		Huang et al., 2018 [36]
**Diet with high DII scores**	**↑ RISK** OR 1.72 (1.26–2.21) in postmenopausal women	Pro-inflammatory diets significantly increase OC risk among post-menopausal women	Yang et al., 2022 [44]
**PID**	**↑ RISK** OR 1.48 (1.03–2.21)		Syed Khaja et al., 2024 [47]
**Aspirin**	**↓ RISK** RR 0.87 (0.80–0.94)		Hurwitz et al., 2022 [51]
**Smoking**	RR 1.44 (1.23–1.67) for invasive mucinous cancer RR 2.09 (1.78–2.46) for mucinous BOT	No association with serous OC	Santucci et al., 2019 [55]

OC = ovarian cancer; BOT = borderline ovarian tumor; HRT = Hormone replacement therapy; DII = Dietary Inflammatory Index; PID = Pelvic inflammatory disease.

## Data Availability

All data relevant to the study are included in the article. All data were extracted from previously published studies; thus, they are publicly available.

## Abbreviations

The following abbreviations are used in this manuscript:

OC	Ovarian Cancer
OR	Odds Ratio
RR	Relative Risk
CI	Confidence Interval
HRT	Hormone Replacement Therapy
PA	Physical Activity
PID	Pelvic Inflammatory Disease
EDCs	Endocrine-Disrupting Chemicals

## References

[1] Webb P.M., Jordan S.J. (2024). Global epidemiology of epithelial ovarian cancer. Nat. Rev. Clin. Oncol..

[2] Arcieri M., Tius V., Filippin S., Aletti G., Lorusso D., Fagotti A., Sehouli J., Zapardiel I., De Iaco P., Scollo P. (2025). Management of Patients with Epithelial Ovarian Cancer: A Systematic Comparison of International Guidelines from Scientific Societies (AIOM-BGCS-ESGO-ESMO-JGSO-NCCN-NICE). Cancers.

[3] Ali A.T., Al-ani O., Al-ani F. (2023). Epidemiology and risk factors for ovarian cancer. Menopausal Rev..

[4] Pietragalla A., Arcieri M., Marchetti C., Scambia G., Fagotti A. (2020). Ovarian cancer predisposition beyond BRCA1 and BRCA2 genes. Int. J. Gynecol. Cancer.

[5] Arcieri M., Tius V., Andreetta C., Restaino S., Biasioli A., Poletto E., Damante G., Ercoli A., Driul L., Fagotti A. (2024). How BRCA and homologous recombination deficiency change therapeutic strategies in ovarian cancer: A review of literature. Front. Oncol..

[6] Staunstrup L.M., Nielsen H.B., Pedersen B.K., Karsdal M., Blair J.P.M., Christensen J.F., Bager C.L. (2019). Cancer risk in relation to body fat distribution, evaluated by DXA-scans, in postmenopausal women—The Prospective Epidemiological Risk Factor (PERF) study. Sci. Rep..

[7] Aguiar M.P., Gomes M.O., Ananias L.F., Silva-Martins L.F., Espindula A.P. (2025). Impact of single nucleotide variants in estrogen genes on ovarian cancer risk: A systematic review and meta-analysis. Endocr. Oncol..

[8] Cramer D.W., Welch W.R. (1983). Determinants of ovarian cancer risk. II. Inferences regarding pathogenesis. J. Natl. Cancer Inst..

[9] Ness R.B., Grisso J.A., Cottreau C., Klapper J., Vergona R., Wheeler J.E., Morgan M., Schlesselman J.J. (2000). Factors Related to Inflammation of the Ovarian Epithelium and Risk of Ovarian Cancer. Epidemiology.

[10] Kotsopoulos J., Gronwald J., McCuaig J.M., Karlan B.Y., Eisen A., Tung N., Bordeleau L., Senter L., Eng C., Couch F. (2020). Breastfeeding and the risk of epithelial ovarian cancer among women with a BRCA1 or BRCA2 mutation. Gynecol. Oncol..

[11] Sung H.K., Ma S.H., Choi J.-Y., Hwang Y., Ahn C., Kim B.-G., Kim Y.-M., Kim J.W., Kang S., Kim J. (2016). The Effect of Breastfeeding Duration and Parity on the Risk of Epithelial Ovarian Cancer: A Systematic Review and Meta-analysis. J. Prev. Med. Public Health.

[12] Collaborative Group on Epidemiological Studies of Ovarian Cancer (2008). Ovarian cancer and oral contraceptives: Collaborative reanalysis of data from 45 epidemiological studies including 23257 women with ovarian cancer and 87303 controls. Lancet.

[13] Arshadi M., Hesari E., Ahmadinezhad M., Yekta E.M., Ebrahimi F., Azizi H., Esfarjani S.V., Rostami M., Khodamoradi F. (2024). The association between oral contraceptive pills and ovarian cancer risk: A systematic review and meta-analysis. Bull. Cancer.

[14] van Bommel M.H.D., IntHout J., Veldmate G., Kets C.M., de Hullu J.A., van Altena A.M., Harmsen M.G. (2023). Contraceptives and cancer risks in BRCA1/2 pathogenic variant carriers: A systematic review and meta-analysis. Hum. Reprod. Update.

[15] Del Pup L., Codacci-Pisanelli G., Peccatori F. (2019). Breast cancer risk of hormonal contraception: Counselling considering new evidence. Crit. Rev. Oncol. Hematol..

[16] Havrilesky L.J., Moorman P.G., Lowery W.J., Gierisch J.M., Coeytaux R.R., Urrutia R.P., Dinan M., McBroom A.J., Hasselblad V., Sanders G.D. (2013). Oral Contraceptive Pills as Primary Prevention for Ovarian Cancer. Obstet. Gynecol..

[17] Hunn J., Rodriguez G.C. (2012). Ovarian Cancer. Clin. Obstet. Gynecol..

[18] Piek J.M., Schauwaert J., Ellis L.B., Zapardiel I., Planchamp F., Koblos K., Kacperczyk-Bartnik J., Bowden S.J., El Hajj H., Grigore M. (2026). Opportunistic Salpingectomy for Prevention of Tubo-Ovarian Carcinoma. JAMA.

[19] Tang Y., Sun H., Fu P., Zhou T., Liu R. (2025). Prophylactic salpingectomy as a preventative strategy for ovarian cancer in the general population: A systematic review and meta-analysis. J. Gynecol. Oncol..

[20] Crum C.P., Drapkin R., Kindelberger D., Medeiros F., Miron A., Lee Y. (2007). Lessons from BRCA: The tubal fimbria emerges as an origin for pelvic serous cancer. Clin. Med. Res..

[21] Wang C., Liang Z., Liu X., Zhang Q., Li S. (2016). The Association between Endometriosis, Tubal Ligation, Hysterectomy and Epithelial Ovarian Cancer: Meta-Analyses. Int. J. Environ. Res. Public Health.

[22] Caruso G., Weroha S.J., Cliby W. (2025). Ovarian Cancer. JAMA.

[23] Xiang H., Wang L., Sun L., Xu S. (2024). The risk of ovarian cancer in hormone replacement therapy users: A systematic review and meta-analysis. Front. Endocrinol..

[24] La Vecchia C. (2017). Ovarian cancer: Epidemiology and risk factors. Eur. J. Cancer Prev..

[25] Barcroft J.F., Galazis N., Jones B.P., Getreu N., Bracewell-Milnes T., Grewal K.J., Sorbi F., Yazbek J., Lathouras K., Smith J.R. (2021). Fertility treatment and cancers—The eternal conundrum: A systematic review and meta-analysis. Hum. Reprod..

[26] Jensen A., Guleria S., Albieri V., Nøhr B., Frederiksen K., Kjær S.K. (2025). Fertility treatment and risk of ovarian cancer in a large nationwide cohort of infertile Danish women. Int. J. Cancer.

[27] Aune D., Rosenblatt D.A.N., Chan D.S.M., Abar L., Vingeliene S., Vieira A.R., Greenwood D.C., Norat T. (2015). Anthropometric factors and ovarian cancer risk: A systematic review and nonlinear dose-response meta-analysis of prospective studies. Int. J. Cancer.

[28] Collaborative Group on Epidemiological Studies of Ovarian Cancer (2012). Ovarian Cancer and Body Size: Individual Participant Meta-Analysis Including 25,157 Women with Ovarian Cancer from 47 Epidemiological Studies. PLoS Med..

[29] Reeves G.K., Pirie K., Beral V., Green J., Spencer E., Bull D. (2007). Cancer incidence and mortality in relation to body mass index in the Million Women Study: Cohort study. BMJ.

[30] Olsen C.M., Nagle C.M., Whiteman D.C., Ness R., Pearce C.L., Pike M.C., Rossing M.A., Terry K.L., Wu A.H., Risch H.A. (2013). Obesity and risk of ovarian cancer subtypes: Evidence from the Ovarian Cancer Association Consortium. Endocr.-Relat. Cancer.

[31] Avgerinos K.I., Spyrou N., Mantzoros C.S., Dalamaga M. (2019). Obesity and cancer risk: Emerging biological mechanisms and perspectives. Metabolism.

[32] Wang L., Zhong L., Xu B., Chen M., Huang H. (2020). Diabetes mellitus and the risk of ovarian cancer: A systematic review and meta-analysis of cohort and case-control studies. BMJ Open.

[33] Tanha K., Mottaghi A., Nojomi M., Moradi M., Rajabzadeh R., Lotfi S., Janani L. (2021). Investigation on factors associated with ovarian cancer: An umbrella review of systematic review and meta-analyses. J. Ovarian Res..

[34] Cao M., Huang Y., Zhou Y., Wang H., Zhang J. (2025). Association between physical activity and gynecological cancers: A meta-analysis of prospective cohort studies. BMC Womens Health.

[35] McTiernan A. (2008). Mechanisms linking physical activity with cancer. Nat. Rev. Cancer.

[36] Huang X., Wang X., Shang J., Lin Y., Yang Y., Song Y., Yu S. (2018). Association between dietary fiber intake and risk of ovarian cancer: A meta-analysis of observational studies. J. Int. Med. Res..

[37] Xu H., Ding Y., Xin X., Wang W., Zhang D. (2018). Dietary fiber intake is associated with a reduced risk of ovarian cancer: A dose-response meta-analysis. Nutr. Res..

[38] Oliverio A., Bruno E., Meli C., Daniele A., Tommasi S., Terribile D., Magno S., Filippone A., Rossi C., Rossi M. (2025). Long-term impact of a Mediterranean diet on BRCA-related cancer risk. Clin. Nutr. ESPEN.

[39] Esposito G., Turati F., Mignozzi S., Parazzini F., Augustin L.S.A., Vitale S., Polesel J., Maso L.D., Negri E., La Vecchia C. (2026). Plant-Based Diets and Ovarian Cancer Risk. Nutrients.

[40] Merritt M.A., Riboli E., Weiderpass E., Tsilidis K.K., Overvad K., Tjønneland A., Hansen L., Dossus L., Fagherazzi G., Baglietto L. (2014). Dietary fat intake and risk of epithelial ovarian cancer in the European Prospective Investigation into Cancer and Nutrition. Cancer Epidemiol..

[41] Khodavandi A., Alizadeh F., Razis A.F.A. (2021). Association between dietary intake and risk of ovarian cancer: A systematic review and meta-analysis. Eur. J. Nutr..

[42] Merritt M.A., Cramer D.W., Missmer S.A., Vitonis A.F., Titus L.J., Terry K.L. (2014). Dietary fat intake and risk of epithelial ovarian cancer by tumour histology. Br. J. Cancer.

[43] Blank M.M., Wentzensen N., Murphy M.A., Hollenbeck A., Park Y. (2012). Dietary fat intake and risk of ovarian cancer in the NIH-AARP Diet and Health Study. Br. J. Cancer.

[44] Yang J., Ma J., Jin Y., Cheng S., Huang S., Wang Y. (2022). Dietary Inflammatory Index and Ovarian Cancer Risk: A Meta-Analysis. Nutr. Cancer.

[45] Augustin L.S.A., Polesel J., Bosetti C., Kendall C.W.C., La Vecchia C., Parpinel M., Conti E., Montella M., Franceschi S., Jenkins D.J.A. (2003). Dietary glycemic index, glycemic load and ovarian cancer risk:a case–control study in Italy. Ann. Oncol..

[46] Restaino S., Pellecchia G., Arcieri M., Pericolini E., Bogani G., Poli A., Paparcura F., Pregnolato S., Armenise D., Frossi B. (2025). The Relationship Between the Vaginal Microbiota and the Ovarian Cancer Microenvironment: A Journey from Ideas to Insights. Cells.

[47] Syed Khaja A.S., Saleem M., Zafar M., Moursi S., Mohammed G.E.Y., Alam Shahid S.M., Hammam S., Moussa S., Alharbi M.S., Alshammari A.N. (2024). Association between pelvic inflammatory disease and risk of ovarian, uterine, cervical, and vaginal cancers—A meta-analysis. Arch. Gynecol. Obstet..

[48] Piao J., Lee E.J., Lee M. (2020). Association between pelvic inflammatory disease and risk of ovarian cancer: An updated meta-analysis. Gynecol. Oncol..

[49] Jonsson S., Jonsson H., Lundin E., Häggström C., Idahl A. (2024). Pelvic inflammatory disease and risk of epithelial ovarian cancer: A national population-based case-control study in Sweden. Am. J. Obstet. Gynecol..

[50] Jonsson S., Jonsson H., Lundin E., Häggström C., Idahl A. (2025). Pelvic inflammatory disease and risk of borderline ovarian tumors: A national population-based case–control study in Sweden. Int. J. Cancer.

[51] Hurwitz L.M., Townsend M.K., Jordan S.J., Patel A.V., Teras L.R., Lacey J.V., Doherty J.A., Harris H.R., Goodman M.T., Shvetsov Y.B. (2022). Modification of the Association Between Frequent Aspirin Use and Ovarian Cancer Risk: A Meta-Analysis Using Individual-Level Data From Two Ovarian Cancer Consortia. J. Clin. Oncol..

[52] Dovnik A., Fokter Dovnik N. (2020). Vitamin D and Ovarian Cancer: Systematic Review of the Literature with a Focus on Molecular Mechanisms. Cells.

[53] Tran B., Jordan S.J., Lucas R., Webb P.M., Neale R. (2012). Association between Ambient Ultraviolet Radiation and Risk of Epithelial Ovarian Cancer. Cancer Prev. Res..

[54] Xu J., Chen K., Zhao F., Huang D., Zhang H., Fu Z., Xu J., Wu Y., Lin H., Zhou Y. (2021). Association between vitamin D/calcium intake and 25-hydroxyvitamin D and risk of ovarian cancer: A dose-response relationship meta-analysis. Eur. J. Clin. Nutr..

[55] Santucci C., Bosetti C., Peveri G., Liu X., Bagnardi V., Specchia C., Gallus S., Lugo A. (2019). Dose–risk relationships between cigarette smoking and ovarian cancer histotypes: A comprehensive meta-analysis. Cancer Causes Control..

[56] Modugno F., Ness R.B., Cottreau C.M. (2002). Cigarette Smoking and the Risk of Mucinous and Nonmucinous Epithelial Ovarian Cancer. Epidemiology.

[57] Micha J.P., Rettenmaier M.A., Bohart R., Goldstein B.H. (2022). Talc powder and ovarian cancer: What is the evidence?. Arch. Gynecol. Obstet..

[58] Berge W., Mundt K., Luu H., Boffetta P. (2018). Genital use of talc and risk of ovarian cancer: A meta-analysis. Eur. J. Cancer Prev..

[59] Wentzensen N., O’Brien K.M. (2021). Talc, body powder, and ovarian cancer: A summary of the epidemiologic evidence. Gynecol. Oncol..

[60] Samtani R., Sharma N., Garg D. (2018). Effects of Endocrine-Disrupting Chemicals and Epigenetic Modifications in Ovarian Cancer: A Review. Reprod. Sci..

[61] Poole E.M., Konstantinopoulos P.A., Terry K.L. (2016). Prognostic implications of reproductive and lifestyle factors in ovarian cancer. Gynecol. Oncol..

[62] Bröckelmann N., Balduzzi S., Harms L., Beyerbach J., Petropoulou M., Kubiak C., Wolkewitz M., Meerpohl J.J., Schwingshackl L. (2022). Evaluating agreement between bodies of evidence from randomized controlled trials and cohort studies in medical research: A meta-epidemiological study. BMC Med..

